# Association of herd BRSV and BHV-1 seroprevalence with respiratory disease and reproductive performance in adult dairy cattle

**DOI:** 10.1186/1751-0147-54-4

**Published:** 2012-01-30

**Authors:** Kerli Raaperi, Stephanie Bougeard, Annely Aleksejev, Toomas Orro, Arvo Viltrop

**Affiliations:** 1 Institute of Veterinary Medicine and Animal Sciences, Estonian University of Life Sciences, Kreutzwaldi 62, Tartu, 51014, Estonia; 2 Department of Epidemiology, Pig, Poultry and Fishes Laboratory, French Agency for Food, Environmental, and Occupational Health Safety (Anses), Zoopole - BP 53, 22440 Ploufragan, France

**Keywords:** Bovine respiratory disease, reproduction, dairy cattle, bovine herpesvirus 1, bovine respiratory syncytial virus

## Abstract

**Background:**

The aim of this study was to detect the associations between bovine herpesvirus 1 (BHV-1) status of a herd and respiratory disease (BRD) occurrence and reproductive performance in pregnant heifers and cows. The association between management-related factors and higher BRD occurrence was also estimated.

**Methods:**

Serum samples, collected from cows and youngstock from 103 dairy cattle herds, were analyzed for antibodies against BHV-1, bovine respiratory syncytial virus (BRSV), bovine viral diarrhoea virus (BVDV), and *Mycoplasma bovis*. A questionnaire was used to collect data concerning herd management factors and reproductive performance, as well as the occurrence of clinical signs of respiratory disease in the last two years, as evaluated by the veterinarian or farm manager. Multiple correspondence analysis (MCA) and logistic regression analysis were performed to identify and quantify the risk factors.

**Results:**

A low to moderate prevalence (1-49%) of BRSV antibodies among youngstock was associated with a high occurrence of respiratory disease (OR = 6.2, p = 0.010) in cows and in-calf heifers. Employees of the farm may participate in the spread of such disease. Larger herd size, loose-housing of cows, housing youngstock separately from cows until pregnancy, and purchasing new animals were factors possibly related to a high occurrence of respiratory disease symptoms in pregnant heifers and cows. The highest risk of abortions (> 1.3%) and increased insemination index (number of inseminations per pregnancy) (> 1.9) occurred in herds with a moderate prevalence of BHV-1 antibodies (1-49%) in cows.

**Conclusions:**

BHV-1 was not associated with acute respiratory disease in adult dairy cattle, however was significantly related to reproductive performance. BRSV possesses the main role in respiratory disease complex in adult dairy cattle.

## Background

Bovine respiratory disease (BRD) incorporates all possible respiratory diseases in cattle and is characterised by abnormal clinical signs of the respiratory tract [1]. Bovine respiratory disease refers to bacterial bronchopneumonia that may be complicated by previous, or concurrent, viral or *Mycoplasma *infection [2]. The principal viruses involved in BRD include bovine herpesvirus 1 (BHV-1), bovine respiratory syncytial virus (BRSV), bovine parainfluenza virus type 3 (PI-3) and bovine viral diarrhoea virus (BVDV) [2]. Despite advances in veterinary medicine, animal husbandry, and animal welfare, respiratory disease among dairy cattle continues to be a major problem in the dairy industry [3]. In addition to enzootic calf pneumonia, outbreaks of respiratory disease in adult animals can have devastating economic outcomes for dairy owners [3].

Many studies have been performed to detect animal-level risk factors for respiratory disease in young calves, whereas the literature concerning BRD in adult dairy cattle is deficient [1,3]. In adult dairy cattle, respiratory disease is less important than mastitis, lameness, or reproductive disorders as a cause of morbidity [2]. According to the Annual Report of the Estonian Animal Recording Centre (EARC, 2009), BRD was the reason for culling dairy cows in 0.7% of cases. According to our experience, in most herds BRD occurs as a sporadic disease in adult dairy cattle. However, epidemic outbreaks occur with high morbidity accompanied with dramatic economic losses due to medication use and discarded milk, as well as cow fatalities.

The subclinical course of BHV-1 infection has been observed after the introduction of the virus to a naive herd [4,5], however high morbidity of BHV-1 outbreaks involving respiratory disease symptoms (lethargy, coughing, conjunctivitis and oculonasal discharge) was seen on a number of occasions [6]. Outbreaks of severe respiratory disease due to bovine respiratory syncytial virus (BRSV) have been observed in dairy herds throughout Sweden, where adult cattle were most severely affected [7]. Risk factors associated with acute bovine respiratory disease, especially with BRSV outbreaks, were larger herd size, as well as the type of the production with a higher risk in dairy herds compared to beef herds [8,9]. Acute BRD has been found to occur mainly during cold months, with an epidemic peak in December [8]. Despite the multifactorial nature of BRD [3], only limited research data is available on herd management-related risk factors for respiratory disease in adult dairy cattle.

Poor fertility is the leading cause of culling cows in Estonia (EACR, 2009). Problems associated with reduced fertility in dairy cattle are related to: diseases of the reproductive tract of the cow, bull fertility, breeding management, and the environment [10], as well as nutrition [11]. Several infectious diseases are related to abortion in cattle, and BHV-1, BVDV and *Neospora caninum *are often diagnosed as causes of abortion in cattle world-wide [12]. However, field studies estimating the effect of BHV-1 on herd level reproductive performance have given contrary results. Previous studies [13,14] found no association between the proportion of calves with antibodies against BVDV or BHV-1 virus and reproductive performance in beef herds. A somewhat higher mean open days period was found in cows that were serologically positive for BHV-1 than in seronegative dairy cows [15], however no decrease in reproduction performance was found to occur during an outbreak of BHV-1 in a dairy herd [4]. To our knowledge no epidemiological studies have been published to identify and quantify the association between herd BHV-1 seroprevalence and farm-level reproductive performance in dairy cattle.

The objective of this study was to ascertain the associations between herd BHV-1 seroprevalence and the occurrence of acute respiratory disease and reproductive performance in adult dairy cattle. The association between management-related factors and higher BRD occurrence was also estimated.

## Methods

### Study design

The survey was conducted between September 2006 and April 2008. The target population in our study was dairy cattle herds with more than 20 cows. Herds were stratified according to number of cows into five classes: 20-49, 50-99, 100-199, 200-399 and > 400 cows. As the main interest of the study was to investigate the influence of BHV-1 infection on herd health, the herds were included in the study depending on their BHV-1 antibody status. For that, results of the bulk tank milk survey of BHV-1 antibodies of all dairy herds in Estonia completed in 2004 were used. Herds were selected randomly from the list by using random number generator. In total, 65 BHV-1 seropositive and 38 BHV-1 seronegative herds, matched by herd size, were selected for the study. Distribution of herds included in the study as well as information about the source population is given in Table 1.

**Table 1:** Number of herds in Estonia in 2007 and study sample size

Herd size	Number of herds in Estonia in 2007	Study sample (BHV-1 antibody positive herds)	Study sample (BHV-1 antibody negative herds)	Study sample in total
20-49	255	9	17	26
50-99	110	7	9	16
100-199	85	14	5	19
200-399	83	18	6	24
≥400	59	17	1	18

Total	592	65	38	103

In each of the selected herds, serum samples from a representative number of randomly selected cows, and youngstock older than six months, were analyzed. In total, 9,637 serum samples were collected. A precise description about the selection of herds, and animals within herds, has been reported in Raaperi et al. [16].

A questionnaire that recorded herd-level data was completed for every herd. The information requested included: herd size, number of livestock units per farm, deployment of the veterinarian and inseminator, frequency of movement of animals between barns, participation in agricultural shows, purchase history of cows, type of housing (cold/warm barn), housing system for cows and youngstock (loose/tied), management of youngstock (separately from cows/contact with cows for some period/in the same barn with cows), use of bull for serving cows and heifers, breed(s) of cattle, grazing management of cows and/or youngstock, vaccination history, whether employees change clothes on the farm, and information about disinfection. In addition, questions were asked relating to the history of the peak occurrence of respiratory disease, within the previous two years, of cows and pregnant heifers. The number of abortions, as well as the herd's average insemination index for cows and heifers for the previous year was recorded if registered in farm. Insemination index is defined as number of inseminations per pregnancy.

### Sample analysis

All serum samples were tested for BHV-1 antibodies using a commercial BHV-1 gB ELISA test kit, HerdChek* (IDEXX, Switzerland) having 100% sensitivity and 99.8% specificity. Suspect antibody test results (samples with blocking % greater than or equal to 45% but less than 55%) were considered as positive in the data analysis.

The herd BVDV status was established by testing up to 10 serum samples from randomly selected animals, at ages from six months up to age at first calving, for BVDV antibodies as recommended by Houe et al. [17]. This enabled detection of a minimum prevalence of 20-28% depending on herd size. The PrioCheck BVDV Ab test kit (Prionics AG, Switzerland) was used for antibody testing. The test has a relative sensitivity and specificity of approximately 98% and 99%, respectively, compared to a virus neutralization test [18].

The herd BRSV status was established by testing up to 20 (depending on herd size) randomly selected serum samples from heifers for BRSV antibodies to allow detection of at least a 15% prevalence of BRSV antibody carriers in the herd at a 95% confidence level assuming 94.6% sensitivity and 100% specificity of the test. For BRSV antibodies, the Svanovir ELISA test (Svanova Biotech AB, Sweden) was used.

Depending on herd size up to 25 heifers and 10 cows were tested for *Mycoplasma bovis *antibodies in each herd. This enabled to detect the prevalence of at least 15% among heifers and 27% among cows with 95% level of confidence. BIO K 260 ELISA test (Bio-X Diagnostics, Belgium) with sensitivity and specificity of 100% in 10% cut-off of optical density of the positive control was used to measure *M. bovis *antibodies.

### Description of the models and categorization of variables

The aim of the first model (Model I) was to clarify the association between herd BHV-1 seroprevalence and respiratory disease occurrence in adult dairy cattle, as well as the detection of management factors associated with higher BRD occurrence. On each farm the veterinarian or farm manager was questioned about the occurrence of clinical signs of respiratory disease, including nasal discharge ("red nose"), respiratory signs (cough, dyspnoea), and lacrimation. The respondents were asked to evaluate the prevalence of these signs among animals of each age group during periods of the highest occurrence of respiratory disease in the previous two years. The scale of the estimation was presented as follows: 1 - no signs or only single cases; 2 - up to 10%; 3 - 10-30%; 4 - over 30%. In order to dichotomize the outcome variables the value of the outcome variables was taken as 1 if more than just a single animals showed the signs concurrently. All of the three variables (see Table 2) were chosen as supplementary variables in Model I when multiple correspondence analysis (MCA) was carried out.

**Table 2:** Descriptive characteristics of the variables included in the models (100 herds in Model I and 77 herds in Model II)

Variable	Definition of the categories of the variable	Number of herds	
		**Model I**	**Model II**

Nasal discharge ("red nose") in cows and/or pregnant heifers	0 - not present at all or was shown only as single cases at some point during the last two years	82	
(NASCOW)	1 - present in more than just single cases at some point during the last two years	18	
Respiratory symptoms (cough, dyspnoea) in cows and/or pregnant heifers	0 - not present at all or was shown only as single cases at some time point during the last two years	80	
(RESCOW)	1 - present in more than just single cases at some point during the last two years	20	
Lacrimation in cows and/or pregnant heifers	0 - not present at all or was shown only as single cases at some point during the last two years	88	
(LACCOW)	1 - present in more than just single cases at some point during the last two years	12	
Respiratory disease occurrence in cows and/or pregnant heifers	0 - less than two respiratory disease symptoms were present in more than a single case at some time during the last two years	81	
(BRDCOW)	1 - at least two respiratory disease symptoms were present in more than a single case at some time during the last two years	19	
Incidence of abortions in the herd	0 - < 1.3% in a herd (median for cut-off value)		37
(ABORT)	1 - ≥ 1.3% in a herd		40
Insemination index for cows and heifers	0 - < 1.9 in a herd (median for cut-off value)		38
(INSIN)	1 - ≥1.9 in a herd		39
Herd size	3 - 20-99 cows	40	25
(hsize)	4 - 100-199 cows	19	17
	5 - 200-399 cows	23	21
	6 - > 400 cows	18	14
Veterinarian an employee of the farm	0 - no	77	
(vetemp)	1 - yes	23	
Does the inseminator give service to other farms	0 - no	28	
(insoth)	1 - yes	72	
Inseminator an employee of the farm	0 - no	56	
(insemp)	1 - yes	44	
Using bull to inseminate heifers	0 - no	61	
(bullh)	1 - yes	39	
Keeping youngstock together with cows	0 - together	51	
(yoco)	1 - in separate building from 6 months until pregnancy	49	
Housing for youngstock	1 - tied	23	
(keyo)	2 - loose	30	
	3 - some period of life tied, some period loose	47	
Housing for cows	1 - tied	71	
(keco)	2 - loose	29	
Has the farm purchased new animals within the last three years (purc)	0 - no 1 - yes	47 53	
BVDV present in the herd	0 - no	77	57
(BVD)	1 - yes (at least one animal tested positive)	23	20
RSV prevalence	0 - negative	46	34
(BRSV)	1 - 1 - 49%	40	33
	2 - ≥50%	14	10
BHV-1 prevalence in cows	0 - negative	37	25
(BHVcow)	1 - 1 - 49%	28	24
	2 - ≥50%	35	28
BHV-1 prevalence in heifers	0 - negative	56	40
(BHVheif)	1 - positive	44	37

As all the respiratory disease symptoms were highly clustered in the MCA analysis (see Results section), one summary variable, describing the level of occurrence of respiratory disease, was created for use in logistic regression analysis. Three variables (RESCOW, NASCOW and LACCOW) were used to create one summary variable. If at least two out of three variables had a value of one, the herd was considered to be in the category of "high occurrence of BRD in cows and/or pregnant heifers" (BRDCOW = 1) (Table 2).

The aim of the second model (Model II) was to detect the linkage between herd BHV-1 seroprevalence and poor reproductive performance in cows and heifers, by taking into account the effect of a possible confounding effect of herd size and other infectious diseases. For that, two outcome variables dichotomized at their median value (1.3% for the proportion of abortions and 1.9 for insemination index) were used in the logistic regression analysis (Table 2).

To obtain more variability in the outcome variables in the smallest category of herd size, herds with 20-49 and those with 50-99 cattle were merged into one smallest herd size category (20-99 cows). All the continuous independent variables were transformed into categorical variables in order to avoid violating the assumption of linearity in logistic regression analysis, and to carry out MCA. The categorization and descriptive statistics of the variables used for the statistical analyses in the models are shown in Table 2.

### Data analysis

In order to select predictor variables to include in the model, univariable logistic regression analysis was performed. Only variables with a p-value lower than 0.2 for any of the outcome variables were included in the data analysis (Table 2). Variables that were not associated with dependent variables in a p-value of ≤0.2 and not included in the model were '*Mycoplasma bovis *prevalence in cows', '*Mycoplasma bovis *prevalence in heifers', 'relocating animals between the barns', 'frequency of overgrouping animals in the farm', 'using bull to inseminate cows', 'grazing youngstock', 'grazing cows' and 'number of livestock units within the farm'. Collinearity among all the outcome and explanatory variables was checked with the chi-square test. Variables 'housing of cows' (tied/loose) and 'barn type' (cold/warm) were highly collinear and had the same explanation so only the former was included in the models having better explaining capacity. Two herds were excluded from the data analysis owing to a large number of missing values. In order to avoid a reduction in the number of herds in the statistical analysis because of the absence of single values in some of the predictor variables, the original dataset was completed using an imputation technique in Stata 11 software (Stata Corporation, Texas, USA). The missing values were replaced using linear regression for multiple imputation for continuous variables and using logistic regression for binary variables (command *mi impute*). For each missing data point, five imputation values were generated and the mean was calculated [19].

Imputed values were created for four herds for the variable "BHV-1 prevalence in heifers" (4%), for six herds for the variables "BVDV prevalence" and "*Mycoplasma bovis *prevalence in heifers" (6%), for eight herds for the variable "BRSV prevalence in heifers" (8%), and for one herd for "*Mycoplasma bovis *prevalence in cows" (1%) in the initial dataset. In Model I, values were missing for some outcome variables for one herd and this herd was excluded from the data analysis. In Model II 24 herds were excluded from the analysis owing to missing values in either of the two outcome variables.

Multiple correspondence analysis (MCA) was used to obtain an overall view of the associations among variable categories in Model I, and to avoid problems arising due to multicollinearity. The test values are considered the standardized coordinates, and are used to interpret the significant variable categories to build each component, *i.e*. with absolute test values higher than the threshold value of 1.96 [20]. The test values are interpreted as the number of standard deviations from the centre of gravity of the analysis. The MCA was performed using XLSTAT (Version 2010.4.01; Addinsoft).

Logistic regression models were built to quantify estimates of the relationships among outcome and predictor variables. All the variables with p-value < 0.2 selected in the univariable logistic regression analysis [21] were included in the multivariable logistic regression model. For the final multivariable model variables with p-value > 0.05 were excluded with backward elimination procedure [21]. The *logit *function of Stata 11 (Stata Corporation, Texas, USA) was applied for logistic regression analysis. The change in regression coefficients was noted to identify important confounding factors (change in regression coefficients between the crude and adjusted value of > 20% [21]), and biologically meaningful interaction were tested. The fit of the model was evaluated with the Hosmer-Lemeshow goodness-of-fit test [21]. There was no indication of lack of fit in the logistic regression models.

## Results

### Risk factors for a high occurrence of respiratory disease symptoms in cows and pregnant heifers (Model I)

The three outcome variables NASCOW, RESCOW and LACCOW were significantly related to each other in the chi-square test. Overall, 33% of the variables related to infections, and 19% of those related to herd management were significantly associated with the outcome variables. Variables describing *Mycoplasma bovis *prevalence in cows and youngstock were not related to any of the outcome variables in the univariable analysis.

**Figure 1:**
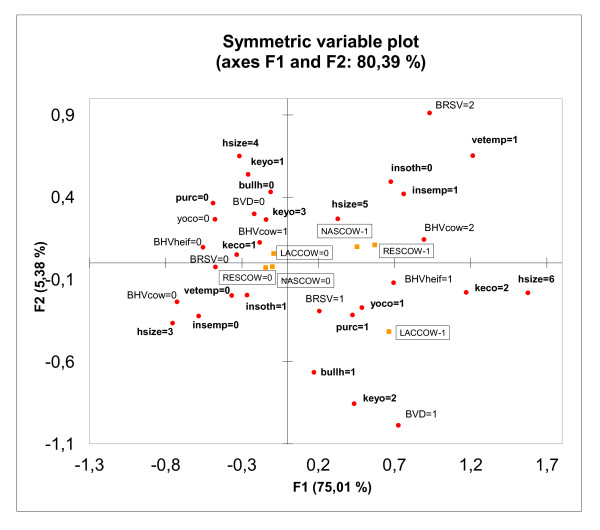
**Graphical display of MCA for high incidence of respiratory disease symptoms in cows and pregnant heifers (100 herds)**.

The total cumulative inertia for the first two axes in the MCA was 80.39% (75.01% and 5.38% for axes 1 and 2). According to the MCA, the management-related variables linked to the first axis, and significantly related to the high occurrence of respiratory disease symptoms, were the largest herd size category (test value 7.35), loose housing of cows (6.20), the veterinarian (6.61) and inseminator (6.60) being the employees of the farm and the latter not providing a service to other farms (4.21), keeping youngstock separately from cows from 6 months until pregnancy (4.77), and purchasing new animals for the herd (4.51) (Figure 1). Infections that were related to a high occurrence of respiratory disease signs in cows and pregnant heifers were: a high prevalence of BHV-1 among cows (6.54) and the presence of BHV-1 among heifers (6.15), the presence of BVDV in a herd (3.95), and a high prevalence of BRSV (3.74).

A low to moderate prevalence of BRSV measured in youngstock (1-49%) was significantly related to a higher occurrence of respiratory disease (OR = 6.2, CI 1.6; 25.0, p = 0.010) among cows and pregnant heifers, according to the results of the logistic regression analysis. Herd size was an insignificant variable in the model, but acted as a confounder and was therefore retained (Table 3).

**Table 3:** Results of logistic regression analysis for risk factors for high occurrence of respiratory disease in pregnant heifers and cows (100 herds)

Risk factor	Herds (n)	OR	p	95% CI
BRSV prevalence in heifers^a^				
0	46	1	-	-
1-49%	40	6.2	0.010	1.6; 25.0
≥50%	14	1.3	0.769	0.2; 8.2
Herd size^b^				
20-99 cows	40	1	-	-
100-199 cows	19	1.3	0.81	0.2; 8.2
200-399 cows	23	2.8	0.160	0.7; 11.8
> 400 cows	18	4.7	0.052	1.0; 22.8

^a^BRSV prevalence in cows p = 0.017, the Wald test

^b^Herd size p = 0.208, the Wald test

### BHV-1 as a risk factor for a high incidence of abortions and high insemination index in breeding animals (Model II)

The results of the logistic regression analysis indicated that, in herds in which BHV-1 is present, among cows the incidence of abortions and the insemination index were higher than those in herds negative for BHV-1. A low to moderate prevalence of BHV-1 among cows (1-49%) was related to the highest risk of a higher incidence of abortions (OR = 7.3, CI 2.0; 26.9, p = 0.003) and an increased insemination index (OR = 5.2, CI 1.5; 18.4, p = 0.010) in a herd. Herd size, as a confounding variable, was also controlled in the model (Table 4).

**Table 4:** Results of logistic regression analysis for risk factors for high abortion and insemination index in cows and heifers (77 herds)

		abortion			insemination index		
Risk factor	Herds (n)	OR	p	95% CI	OR	p	95% CI
							
BHV-1 prevalence in cows^a^							
0	25	1	-	-	1	-	-
1-49%	24	7.3	0.003	2.0; 26.9	5.2	0.01	1.5; 18.4
≥50%	28	4.6	0.022	1.2; 16.7	3.4	0.056	1.0; 12.3
Herd size^b^							
20-99 cows	25	1	-	-	1	-	-
100-199 cows	17	1.2	0.754	0.3; 5.0	0.8	0.725	0.2; 3.0
200-399 cows	21	1.7	0.447	0.4; 6.5	1.2	0.83	0.3; 4.2
> 400 cows	14	0.3	0.165	0.1; 1.6	0.9	0.944	0.2; 4.2

		^a^BHV-1 in cows p = 0.009, the Wald test			^a^BHV-1 cows p = 0.033, the Wald test		
		^b^Herd size p = 0.193, the Wald test			^b^Herd size p = 0.957, the Wald test		

## Discussion

### Risk factors for a high occurrence of respiratory disease symptoms in cows and pregnant heifers

Three symptoms most commonly related to respiratory disease were asked to evaluate by the respondents. As relatively small number of herds had values equal or higher than 'up to 10%', the variables were dichotomised separating herds with high or low occurrence of that symptom. In the graphical display of MCA all three respiratory disease symptoms were closely linked meaning that those were present concurrently in most of the herds. This encouraged us to create one summary outcome variable describing the occurrence of respiratory disease. We find that asking more precise preliminary information from the respondents and controlling the explanation capability of the variables before combining those into one outcome variable has decreased the recall bias and gives easily interpretable results.

BHV-1, BVDV and BRSV are associated with a high occurrence of respiratory disease in Estonian adult dairy cattle, according to the results of the MCA. A high prevalence of BRSV (≥50%) was associated with a high occurrence of respiratory disease symptoms in cows and pregnant heifers in the MCA. When combining these three BRD symptoms into one outcome variable in logistic regression analysis, a low to moderate prevalence of BRSV (1-49%) among youngstock was significantly associated with a high occurrence of respiratory disease among cows and pregnant heifers. This discrepancy may arise from the fact that relatively small number of herds (n = 14) belong to the highest BRSV prevalence group. This may result in larger standard errors of the estimates in the logistic regression analysis affecting also the p-value of the predictor. MCA on the other hand is not as sensitive to sample size as conditional methods. Sampling antibodies from a small number of young animals that have lost maternal immunity indicates the recent spread of infection [22]. However, some studies have shown that outbreaks of acute respiratory disease associated with BRSV in fully susceptible populations affect adult cattle, pregnant or newly calved cows, most severely [7,9]. Thereafter the disease remains endemic, manifesting itself among younger animals that serve as sentinels [7]. Given that the signs of respiratory disease reported in this study were those associated with the occurrence of respiratory disease in the previous two years, cows and pregnant heifers might have experienced disease caused by BRSV some time previously, following the active spread of the virus among youngstock detected in this study at the time of testing. In a severe outbreak of BRSV in Sweden it was found that concurrent infection with other viruses may affect the expression of disease [7]. In addition to BRSV BHV-1 and BVDV were associated with higher occurrence of BRD in MCA. As associations between variables are not adjusted for the effects of other variables with this method we can't state that BHV-1 and BVDV are direct risk factors for BRD. However, apparent bivariate association between these variable gives a reason to suggest that BHV-1 and BVDV may participate in the expression of BRD rather as contributing agents.

Large herd size has been found to be a risk factor for the high occurrence of respiratory disease in many studies [8,9,23]. Elvander [7] has shown that BRSV spreads rapidly within the herd. In larger dairy herds there are more numerous between-animal contacts [8], increased inter- and intra-farm traffic by farm employees such as veterinarians and AI-technicians [8,9,16,23] as well as potential higher animal densities [8] allowing the more efficient spread of infectious agents. The number of animals susceptible to infections in large herds is also higher than in small herds contributing maintenance of infections within a herd over extended periods [23].

MCA has spotlighted several other management practices as possible risk factors for BRD in adult dairy cattle. Although these associations are not conclusive due to the limitations of MCA, they are worthwhile to mention here as factors likely contributing to the disease and requiring attention. First, loose housing of cows was associated with a higher level of BRD in cows and pregnant heifers. We may suggest that more direct contacts between the animals, and the frequent regrouping of animals in loose housing barns, create greater possibilities for the direct transmission of the infectious agents over the whole farm.

Second, housing youngstock in a separate building from six months of age until service was associated with a higher occurrence of BRD. In order to maintain an immunizing infection, the susceptible pool must be replenished via recruitment [24]. Depending on the pattern of infectious disease epidemiology within the herd, commingling animals with different immunity status to specific infections may predispose the active circulation of the virus.

Newly purchased animals can be the source of BRSV infection, which was confirmed in a Swedish study in which outbreaks of BRSV occurred most often after the introduction of purchased animals [7].

High occurrence of respiratory disease was present in 19% of herds included in this study. Due to sporadic nature of the disease the sample size evaluating risk factors for high occurrence of BRD in adult dairy cattle should be larger. Therefore the results of this study give first insight about the risk factors associated with the disease and some factors might have been missed as significant influencing factors.

### BHV-1 as a risk factor for a high incidence of abortions and increased insemination index in breeding animals

BHV-1 increases the risk of a herd having a poor reproductive performance. We can suppose that in herds with a moderate BHV-1 seroprevalence among cows, the level of infection has been low for some time, which enables a susceptible population to evolve, and it is these herds that are most vulnerable to active virus spread and a higher level of endemic abortions. Abortions due to BHV-1 generally occur between four and eight months of gestation, however the infection can also result in early embryonic death [25] resulting in a higher insemination index. As reproduction values were registered retrospectively and antibodies present to BHV-1 reflect virus spread in the past, it is not possible to draw exact cause-effect relationships. Therefore it is possible that poor fertility as well as spread of BHV-1 is influenced by another common factor e.g. poor management practice. A more thorough study involving farm management practices in addition to infections should be conducted.

Association between BHV-1 and fertility of cows and pregnant heifers has been evaluated previously. In field studies, where the course of BHV-1 infection in previously naive herds was recorded, neither an increase in abortion incidence nor a lower proportion of successful inseminations was found [4,5,26]. The impact of BHV-1 on reproduction performance has also been evaluated indirectly. No associations between the proportion of calves with antibodies against IBR virus and incidence of abortions, stillbirths, calf death, nor non-pregnancy were ascertained [14]. However a 17 day longer period for successful conception was required for BHV-1 seropositive rather than seronegative cows [15]. Differences in the results between studies may arise from differences in study design and discrepancies in other herd characteristics as well as the BHV-1 strain involved.

BVDV was also included in this study as a possible confounder for BHV-1, or directly related to fertility. The presence of BVDV was not associated with reproductive performance in this study. A negative impact of BVDV on reproductive efficiency of the herd has likewise not been found in previous studies [14,27,28], however significant relationships might have been missed in our study because of the relatively small sample size (only 20 BVDV-positive herds). As *Neospora caninum *is the pathogen often diagnosed as a cause of abortions in cattle [12] and related to a higher risk of non-pregnancy and abortions [13], it might be important to investigate to obtain more accurate results.

### Contribution of statistical methods

Correspondence analysis is an exploratory multivariate technique for the graphical and numerical analysis [29] designed to analyse the relationships among a set of categorical variables [21]. The result is a scatterplot which identifies clusters of predictors that are closely associated, with clusters farther from the intersection of the axes having stronger associations. The values of the outcome variables were also projected on the same axes to determine which clusters of predictor variable values were associated with the outcomes of interest [21]. A high level of multicollinearity was found using chi-square analysis. Multiple correspondence analysis can be an alternative tool when analysing relationships between different variables in terms of multicollinearity [30]. MCA showed that all supplementary variables belonging to the same category were highly related; this result was used as a justification to generate one outcome variable describing high/low respiratory disease occurrence in cows and pregnant heifers. The latter variable was used as an outcome variable in the logistic regression analysis. MCA also gave an insight into associations between outcome and predictor variables, as well as associations between different predictor variables. As these associations are not adjusted for other variables in the analysis we discuss those as possibly relevant associations. MCA is used as a preliminary analysis to have a clear and reliable view of the variable links in presence of multicollinearity. Our final conclusions confirming the risk factor status of each variable rely on the results of the logistic regression analysis.

## Conclusions

The results of this study demonstrate the significant role of BRSV in the aetiology of BRD in Estonian adult dairy cattle. The presence of neither BHV-1 nor BVDV were associated with acute respiratory disease in adult dairy cattle, however these may not be excluded as possible contributors to the disease. Therefore, precautions to prevent the introduction of the BRS virus into herds should be implemented. In order to reduce the incidence of BRD in dairy cattle, on-farm biosecurity measures may be important as well to prevent human-mediated spread of the infections. As indicated in MCA direct animal contacts in loose-housing systems may increase the occurrence of BRD in cows and pregnant heifers, perhaps *via *increased virus transmission. In order to reduce the circulation of infectious agents in the system, animals should be checked for clinical signs of respiratory disease continuously, and those with symptoms separated immediately from healthy animals. If youngstock and cows are kept in separate units, the aetiology of respiratory disease among both animal groups should be ascertained, followed by the application of specific control measures in order to avoid unprotected animals becoming infected.

In herds with poor reproductive performance, BHV-1 should be considered as one of the infectious risk factors, and the eradication of this virus may improve the reproductive performance of the herd.

## Competing interests

The authors declare that they have no competing interests.

## Authors' contributions

KR was involved in the developing the study design, performing the field study, analysing data and writing the manuscript. SB helped analysing data and revising the manuscript. AA participated in designing the study and performing sample analysis and storage of samples. TO and AV applied for funding, attended in designing the study, analysing data and drafting the manuscript. All authors have read and approved the final version of the manuscript.

## References

[B1] Van der Fels-Klerx HJ Martin SW Nielen M Huirne RBM Effects on productivity and risk factors of Bovine Respiratory Disease in dairy heifers; a review for the Netherlands Neth J Agr Sci 2002 50 27 45

[B2] Callan RJ Garry FB Biosecurity and bovine respiratory disease Vet Clin North Am Food Anim Pract 2002 18 57 77 10.1016/S0749-0720(02)00004-X 12064169 PMC7126375

[B3] Gorden PJ Plummer P Control, Management, and Prevention of Bovine Respiratory Disease in Dairy Calves and Cows Vet Clin North Am Food Anim Pract 2010 26 234 259 10.1016/j.cvfa.2010.03.004 PMC7135383 20619182

[B4] Hage JJ Schukken YH Dijkstra T Barkema HW van Valkengoed PHR Wentink GH Milk production and reproduction during a subclinical bovine herpesvirus 1 infection on a dairy farm Prev Vet Med 1998 34 97 106 10.1016/S0167-5877(97)00088-3 9604259

[B5] Pritchard GC Banks M Vernon RE Subclinical breakdown with infectious bovine rhinotracheitis virus infection in dairy herd of high health status Vet Rec 2003 153 113 117 10.1136/vr.153.4.113 12918828

[B6] Wiseman A Msolla PM Selman IE Allan EM Pirie HM Clinical and epidemiological features of 15 incidents of severe infectious bovine rhinotracheitis Vet Rec 1980 107 436 441 10.1136/vr.107.19.436 7456295

[B7] Elvander M Severe respiratory disease in dairy cows caused by infection with bovine respiratory syncytial virus Vet Rec 1996 138 101 105 10.1136/vr.138.5.101 8650902

[B8] Gay E Barnouin J A nation-wide epidemiological study of acute bovine respiratory disease in France Prev Vet Med 2009 89 265 271 10.1016/j.prevetmed.2009.02.013 19297044 PMC7126910

[B9] Norström M Skjerve E Jarp J Risk factors for epidemic respiratory disease in Norwegian cattle herds Prev Vet Med 2000 44 87 96 10.1016/S0167-5877(99)00113-0 10727746

[B10] Radostits OM Merchant T, Hund R Managing Reproductive Efficiency in Dairy Herds Herd Health: food animal production medicine 2001 3 Pennsylvania: Saunders (W.B.) Co Ltd 255 289

[B11] Roche JF The effect of nutritional management of the dairy cow on reproductive efficiency Anim Reprod Sci 2006 96 282 296 10.1016/j.anireprosci.2006.08.007 16996705

[B12] Anderson ML Infectious causes of bovine abortion during mid- to late-gestation Theriogenology 2007 68 474 486 10.1016/j.theriogenology.2007.04.001 17467787

[B13] Waldner CL Serological status for *N. caninum*, bovine viral diarrhea virus, and infectious bovine rhinotracheitis virus at pregnancy testing and reproductive performance in beef herds Anim Reprod Sci 2005 90 219 242 10.1016/j.anireprosci.2005.03.017 15893892

[B14] Waldner CL Kennedy RI Associations between health and productivity in cow-calf beef herds and persistent infection with bovine viral diarrhea virus, antibodies against bovine viral diarrhea virus, or antibodies against infectious bovine rhinotracheitis virus in calves Am J Vet Res 2008 69 916 92 10.2460/ajvr.69.7.916 18593246

[B15] Ata A Kale M Yavru S Bulut O Buyukyoruk U The effect of subclinical bovine herpesvirilis 1 infection on fertility of cows and heifers Acta Vet (Beogr) 2006 56 267 273 10.2298/AVB0603267A

[B16] Raaperi K Nurmoja I Orro T Viltrop A Seroepidemiology of bovine herpesvirus 1 (BHV1) infection among Estonian dairy herds and risk factors for the spread within herds Prev Vet Med 2010 96 74 81 10.1016/j.prevetmed.2010.06.001 20598386

[B17] Houe H Lindberg A Moennig V Test strategies in bovine viral diarrhea virus control and eradication campaigns in Europe J Vet Diagn Invest 2006 18 427 436 10.1177/104063870601800501 17037609

[B18] Kramps JA van Maanen C van de Wetering G Stienstra G Quak S Brinkhof J Rønsholt L Nylin B A simple, rapid and reliable enzyme-linked immunosorbent assay for the detection of bovine virus diarrhoea virus (BVDV) specific antibodies in cattle serum, plasma and bulk milk Vet Microbiol 1999 64 135 44 10.1016/S0378-1135(98)00265-X 10028168

[B19] Allison PD Missing Data 2002 London: SAGE Publications

[B20] Lebart L Greenacre M, Blasius J Validation Techniques in Multiple Correspondence Analysis Multiple Correspondence Analysis and Related Methods 2006 Chapman & Hall 179 195

[B21] Dohoo I Martin W Stryhn H Veterinary Epidemiologic Research 2009 Charlottetown: VER Inc

[B22] Ohlson A Emanuelson U Tråvén M Alenius S The relationship between antibody status to bovine corona virus and bovine respiratory syncytial virus and disease incidence, reproduction and herd characteristics in dairy herds Acta Vet Scand 2010 52 37 10.1186/1751-0147-52-37 20525326 PMC2891787

[B23] Gulliksen SM Jor E Lie KI Løken T Åkerstedt J Østerås O Respiratory infections in Norwegian dairy calves J Dairy Sci 2009 92 5139 5146 10.3168/jds.2009-2224 19762832 PMC7126448

[B24] Keeling M Rohani P Modeling Infectious Diseases in Humans and Animals 2008 Princeton: Princeton University Press

[B25] Givens MD Marley MSD Infectious causes of embryonic and fetal mortality Theriogenology 2008 70 270 285 10.1016/j.theriogenology.2008.04.018 18502494 PMC7103133

[B26] Cook N Combined outbreak of the genital and conjunctival forms of bovine herpesvirus 1 infection in a UK dairy herd Vet Rec 1998 143 561 562 10.1136/vr.143.20.561 9854320

[B27] Kale M Ata A Yavru S Yapkic O Bulut O Gulay MS The effect of infection with bovine viral diarrhea virus on the fertility of cows and heifers Acta Vet (Beogr) 2006 56 467 477 10.2298/AVB0606467K

[B28] Obando C Ocanto D Hidalgo M Duran JRY Effect of infectious bovine rhinotracheitis virus and bovine viral diarrhea virus infections on reproduction in a non-vaccinated cattle herd Rev Cient (Maracaibo) 2004 14 207 212

[B29] Blasius J Greenacre M Greenacre M, Blasius J Correspondence Analysis and Related Methods in Practice Multiple Correspondence Analysis and Related Methods 2006 Chapman & Hall 3 40

[B30] Dohoo IR Ducrot C Fourichon C Donald A Humik D An overview of techniques for dealing with large numbers of independent variables in epidemiologic studies Prev Vet Med 1996 29 221 239 10.1016/s0167-5877(96)01074-4 9234406

